# Sustainable Sourcing of Global Agricultural Raw Materials: Assessing Gaps in Key Impact and Vulnerability Issues and Indicators

**DOI:** 10.1371/journal.pone.0128752

**Published:** 2015-06-11

**Authors:** Nathaniel P. Springer, Kelly Garbach, Kathleen Guillozet, Van R. Haden, Prashant Hedao, Allan D. Hollander, Patrick R. Huber, Christina Ingersoll, Megan Langner, Genevieve Lipari, Yaser Mohammadi, Ruthie Musker, Marina Piatto, Courtney Riggle, Melissa Schweisguth, Emily Sin, Sara Snider, Nataša Vidic, Aubrey White, Sonja Brodt, James F. Quinn, Thomas P. Tomich

**Affiliations:** 1 Agricultural Sustainability Institute, University of California Davis, Davis, California, United States of America; 2 Information Center for the Environment, University of California Davis, Davis, California, United States of America; Northwest A&F University, CHINA

## Abstract

Understanding how to source agricultural raw materials sustainably is challenging in today’s globalized food system given the variety of issues to be considered and the multitude of suggested indicators for representing these issues. Furthermore, stakeholders in the global food system both impact these issues and are themselves vulnerable to these issues, an important duality that is often implied but not explicitly described. The attention given to these issues and conceptual frameworks varies greatly—depending largely on the stakeholder perspective—as does the set of indicators developed to measure them. To better structure these complex relationships and assess any gaps, we collate a comprehensive list of sustainability issues and a database of sustainability indicators to represent them. To assure a breadth of inclusion, the issues are pulled from the following three perspectives: major global sustainability assessments, sustainability communications from global food companies, and conceptual frameworks of sustainable livelihoods from academic publications. These terms are integrated across perspectives using a common vocabulary, classified by their relevance to impacts and vulnerabilities, and categorized into groups by economic, environmental, physical, human, social, and political characteristics. These issues are then associated with over 2,000 sustainability indicators gathered from existing sources. A gap analysis is then performed to determine if particular issues and issue groups are over or underrepresented. This process results in 44 “integrated” issues—24 impact issues and 36 vulnerability issues —that are composed of 318 “component” issues. The gap analysis shows that although every integrated issue is mentioned at least 40% of the time across perspectives, no issue is mentioned more than 70% of the time. A few issues infrequently mentioned across perspectives also have relatively few indicators available to fully represent them. Issues in the impact framework generally have fewer gaps than those in the vulnerability framework.

## Introduction

Understanding the sustainability implications of all stages of global supply chains has become an important consideration of sustainability efforts by both public and private sector institutions [1–3]. In the case of food products, the largest sustainability impacts often happen during the farming and production of agricultural raw materials, not necessarily in the transport or manufacturing of the final product [4–6]. This understanding has motivated food companies to more precisely define what issues should be considered to improve the sustainability of their sourcing decisions and what metrics are available to measure their current state and future progress [7,8].

Yet the choice of particular issues and indicators that are used to define and measure sustainability can depend greatly upon one’s perspective within the food system. For instance, recent sustainability efforts of food companies have been motivated by increased awareness of indirect impacts of particular environmental issues, which is not surprising given that the concept of sustainability grew from environmentally-focused roots [9–11]. Yet it has been well-established and accepted that in addition to environmental issues, a comprehensive definition of corporate sustainability must also include economic and social issues [12–14]. A number of third-party initiatives [15–17] have brought attention to this broader conceptualization, but there is still no community-wide consensus regarding a comprehensive set of issues that must at least be considered when making a sustainability claim [18].

It is also likely that that some of the issues encompassed by these corporate perspectives are quite different from the issues considered important by the international development community, farmers in developing countries, or scientists and academics. For instance, numerous sustainability initiatives and international organizations have developed in-depth indices and indicator lists for measuring agricultural sustainability issues, addressing environmental, social, and economic issues with various degrees of detail and coverage. Some of these issue and indicator choices have been developed alongside goals at a broad policy level (such as the Millennium Development Goals [19] and the planned Sustainable Development Goals [20]) and others as specific standards for crop certification or issue-specific monitoring (such as the Stewardship Index for Specialty Crops [21]). Such issue and indicator choices may remain biased towards one’s perspective, and without considering multiple perspectives simultaneously, key issues or useful indicators may be easily missed.

Furthermore, consideration of the environmental, social, and economic impacts of a supply chain is only one way to account for sustainability. The vulnerability of a supply chain to particular issues–such as diseases, water scarcity, or climate change–has become another important lens for understanding sustainability in food systems [22–24]. Knowing which issues have the potential to make supply chains more vulnerable and finding indicators to measure and track them is important for farmers, governments, consumers, and food companies alike. And such vulnerability issues may be quite different than those that motivate action to reduce sustainability impacts.

As a result of these varied and complex representations, decision-makers in the food system lack a comprehensive list of the sustainability issues to consider when trying to define and measure sustainability in their supply chains. Instead, they are challenged by a barrage of issues and indicators, many of which overlap and conflict with each other, making it difficult to strategize and consider tradeoffs simultaneously. Moreover, since different stakeholders have different perspectives, simply selecting which issues to consider, much less the vocabulary and metrics by which to describe and judge them, becomes a complex problem. These challenges are compounded for those actors working with multiple crop and regional contexts, since addressing the sustainability of each commodity chain in isolation can be both costly and time-consuming.

To address the absence of such a list, this study presents an integrated network of the various sustainability issues and indicators that any actor could use to make a comprehensive claim about the sustainability of a given agricultural raw material supply chain. This network is generated by cataloguing, collating, and linking a set of diverse sustainability issues, taken from the written communications of three distinct perspectives: global sustainability assessments, private food companies, and academic studies on local community livelihoods. This network is given further context by associating each issue with two potentially relevant frameworks for defining sustainable sourcing: impacts and vulnerabilities. Agricultural raw material sourcing decisions may impact certain issues while other issues may make supply chains more vulnerable–and often both. The network is completed by linking each issue with any of the numerous sustainability indicators that have been proposed by international organizations and initiatives to measure that issue for a given context.

In addition to generating this list, the issue and indicator network is used to calculate the issues that are considered the most and the least often from various perspectives, and which issues have the most and relatively fewest indicators available to represent them. These “gaps” highlight issues that may require additional attention from a given perspective or from the sustainability community as whole. For instance, we hypothesized that food companies would tend to emphasize environmental sustainability issues since some of their recent sustainability efforts are often motivated by increased awareness of indirect environmental impacts stemming from increased use of such methods as life-cycle analysis and footprint calculations [3,16,18]. On the other hand, we thought global sustainability assessments would be more balanced across environmental, economic, and social impacts given their sustainable development framing [19,25] and that the livelihoods perspective would probably put more emphasis on social and political issues, particularly from a vulnerability framing, due to an emphasis on local food security [26–29]. We also posited that more indicators would be found that measure economic and environmental issues than social issues, since the latter tend to be harder to quantify [30,31]. For the same reason, we thought that indicators would more often be used to measure impacts compared to vulnerabilities.

The next section presents the data and methods used to first create the issue and indicator network and then perform the gap analysis that tested our hypotheses. To be clear, the authors of this study do not choose what is considered a sustainability issue or how sustainability is defined; as we will show, sustainability is defined by the set of issues harvested from the individual communications of practitioners from the various perspectives. These communications highlight the issues that are deemed, by each user, to be important sustainability issues. The data and methods section explains how we selected these communications, how we recorded the issues, how we used a controlled vocabulary to make the issues comparable, how we organized those integrated issues into types and frameworks to better grasp the context of the issues, and ultimately how the gap analysis was performed to show where the definition of sustainability is the same and where it is different. The results of the gap analysis are then presented along with some concluding thoughts and future directions for this research.

## Data and Methods

### Ethics Statement

As this study did not obtain information about living individuals, no human research was undertaken and no IRB approval required as defined by U.S. Department of Health and Human Services [32]. Stakeholder advisors verbally consented that anonymized guidance (using Chatham House Rules) could be used and published as part of this study. All data collected and created by this study is open access, see Information B in S1 File.

### An integrated list of sustainability issues

A global set of possible sustainability issues is a prerequisite for identifying the subset of issues relevant to the sustainable sourcing of agricultural raw materials. This global set was identified through text searches of key public communications from three different stakeholder “perspectives”. Two of these perspectives are “top-down”: a public policy perspective, represented by global sustainability assessments, and a private sector perspective, represented by the public communications of food companies. The third perspective is “bottom-up”, represented by conceptual frameworks of sustainable livelihoods from the academic literature [33]. All issues directly identified in the text of these communications are called the “verbatim” issues and the methods for identifying these verbatim issues for each perspective are described below. See Information B in S1 File for more information and datasets regarding the verbatim issues for each of the three perspectives.

#### Perspective 1: Global Assessments

Many initiatives to improve sustainability around the world have emerged since sustainable development was identified as a policy goal [19,34,35]. Initiatives often report upon specific issues they find important using assessments on the state of sustainability [36]. Such assessments were found by performing a literature search in Google Scholar and Web of Science (key words: global, sustainability, ecosystem assess*, evaluate*, monitor*) and then vetted using four key criteria: assessments that focused on multiple sustainability dimensions (environmental, social, economic); that were designed using scientifically-based processes; that were flexible in their application to various farming systems and geographical scales; and that were global in scope. These four criteria ensured that each assessment was salient, legitimate, and transparent while also fitting the scale and scope of this perspective. Fifteen such assessments were found met these criteria and were selected for analysis by this study (see Table A in S1 File). Major reports from these fifteen assessments were then examined for any sustainability issues identified as important by the initiative and presented in the text, figures or tables [17,24,37–50]. These issues were recorded verbatim (see S1 Dataset).

#### Perspective 2: Food Companies

To record the issues deemed important from a private sector perspective, our team examined all the publically available internet-based communications of the ten largest global food manufacturers (by global sales): Nestle, Kraft Foods, Mars, Unilever, PepsiCo, Danone, Kellogg’s, General Mills, Heinz, and Campbell’s Soup Company. These communications include any sustainability or corporate social responsibility reports along with additional acknowledgments, commitments, accomplishments, or assessments of self-identified sustainability practices included on their corporate websites in 2012. Such websites often devote one or more sections to issues of sustainability, and may refer directly to “sustainability” or use different terminology such as “responsibility”, “our impacts”, “our world”, etc. Nearly 500 such communications were identified and analyzed (see S2 Dataset). Any statement about corporate sustainability practices in these communications was captured, from general statements and acknowledgments of impacts to specific commitments on particular issues. The verbatim sustainability issues within with these statements were captured as well.

#### Perspective 3: Livelihoods Frameworks

To provide a perspective that is focused on producers and rural communities, we surveyed the academic literature describing frameworks of sustainable livelihoods. These studies identify sets of issues that are deemed necessary for achieving and maintaining sustainable livelihoods at the community scale. Framework identification involved searches for the key terms ‘sustainable livelihood’, ‘food and nutrition system’, and ‘food security’ in search engines including Google Scholar and Web of Knowledge. Selection criteria included those papers or publications that: contained a depiction of a conceptual framework; were relevant to agriculture, food systems or food security; were not limited to a specific location or context; and met credibility standards including publication in the peer-reviewed literature or institutional affiliation. Twelve studies were identified that matched these criteria [28,51–61]. Sustainability issues explicitly mentioned in diagrams depicting the conceptual frameworks were recorded verbatim (see Table B in S1 File).

#### Integrating Issues Across Perspectives Using AGROVOC

The outcome of this process was similar yet distinct lists of issues for the three perspectives. Some issues were very broad, others very narrow, and often each perspective used different words to represent the same issue. Therefore, these three lists of verbatim issues were merged, creating an “integrated” list of issues–a list with the same vocabulary regardless of the source. For instance, the global assessment SAFA [50] uses the term “Investment”, the livelihood study by Pender *et al*. [52] uses the term “Access to Finance”, and General Mills uses the phrase “Providing Interest-Free Loans”. All three of these terms were mapped to the new integrated issue Finance, allowing for comparison and contrast across the three perspectives. This integrated list of issues was created using terms from the AGROVOC thesaurus, a widely used controlled vocabulary created by the FAO to standardize agricultural terminology [62]. The verbatim issues from the three perspectives were mapped to this integrated list, with the scale and scope of language adjusted as necessary (see S3 Dataset). New terms were used if no term existed in AGROVOC that adequately integrated a group of verbatim issues.

#### Organizing Integrated Issues by Capital Groups

Next, issues with similar characteristics were put into groups, which allowed the discernment of certain types of issues are particularly underrepresented. Although sustainability issues are commonly categorized in three types–economic, environmental, and social [12–14]–a finer classification can provide more nuance to describe particular issues. The sustainable livelihoods and food security literature provides such a classification that distinguishes six different types of “capital”, which are defined as the endowments and assets available to a given population [63–66]. For instance, from the perspective of a household, financial capital is the available monetary assets while natural capital is the available natural resource endowments (such as land, water, biomass, etc.).

Assessing these six types—financial, human, natural, physical, political, and social–it was discovered that issues could be combined into four groups based upon the units of measurement of these capital types. These four groups–hereafter referred to as the “capital groups”–are as follows: human issues that are measured on a per capita basis, natural issues that are measured using biophysical units, physical and financial issues that are measured in monetary units, and social and political issues that are measured in terms of whole communities or societies of people. For instance, the human issue Nutritional Status can be measured in nutrients per person, the natural issue Water can be measured in cubic meters, the physical issue Physical Infrastructure can be measured in dollars, and the social issue Educational Resources can be measured as the percentage of educated individuals in a given community.

#### Integrated Issues are Comprised of Component Issues

Many of the integrated issues chosen from AGROVOC are fairly broad (for instance Land and Soil or Public Health). Therefore, a list of key “component” issues was identified to complement and further describe the integrated issues. For instance, the integrated issue Water is comprised of a number of component issues including Water Scarcity, Water Availability, Water Quality, etcetera. Terms for component issues were taken from AGROVOC when possible, but were supplemented by additional terminology from the Linked Data Service, a controlled vocabulary maintained by the Library of Congress [67]. Where possible, the same language as the verbatim issues was used. The indicator dataset was also cross-referenced to ensure all indicators had a directly relevant component issue, even if this meant creating a new term that did not exist as a verbatim issue or in the AGROVOC/Linked Data Service vocabularies.

See S4 Dataset information list of all component issues and how they link to the integrated issues. Note that these components are not necessarily narrower or broader than the integrated issue, but each is a distinctly important piece of the integrated issue. For instance, Food Security was identified as a component of the integrated issue Air & Climate, but it is arguably broader than this issue as well. On the other hand, the component issue Climate Change is obviously narrower than Air & Climate.

Each component issue was then linked to any additional integrated issues that it could help describe. For most components, this meant linking to multiple integrated issues. For instance, although Water Quality is obviously a component of Water, it is also a component of five other integrated issues, including Public Health, and Wastes & Pollution.

### Two sustainable sourcing frameworks: impact and vulnerability issues

After integrating our comprehensive list of sustainability issues, the issues particular to sustainable sourcing in agriculture and the food industry were identified. Not all sustainability issues are relevant to every sustainable sourcing decision-makers and stakeholders trying to improve their sourcing practices, and different issues are important for different reasons. This study employed conceptual frameworks–formal representations of the structure and relationships between concepts and issues [66]–to identify and highlight the importance of both sustainability impacts as well as supply-chain vulnerabilities. Issues that fall within the bounds of a given framework are issues that should be considered when trying to either mitigate impacts or create increasingly resilient and adaptive supply chains.

#### Impact issues

If the sourcing of agricultural raw materials could have a direct impact on a given issue (and hence could also directly mitigate that issue), it was placed in the impact framework. For example, a sourcing decision can directly impact Climate Change and can be directly mitigated by working with farmers to reduce greenhouse gas emissions. The entire list of integrated issues was systematically assessed one-by-one according to this criterion, resulting in a comprehensive set of issues that one could use to define the sustainability impacts of sourcing agricultural raw materials.

#### Vulnerability issues

If the sourcing of agricultural materials is directly vulnerable to a given issue (and hence can be directly made more resilient), it was placed in the vulnerability framework. For example, sourcing of agricultural materials can be vulnerable to Climate Change and can also be made more resilient through the adoption of climate-robust crop varieties. Each integrated issue was assessed according to whether or not it met this criterion, resulting in a comprehensive set of issues that one could use to define the vulnerabilities of sourcing agricultural raw materials sustainably. As demonstrated by the example issue Climate Change, integrated issues can be in both the impact and vulnerability framework, although some fall in only one or the other.

### Creating a Database of Sustainability Indicators

A list of existing sustainability indicators was compiled to measure and represent the issues. All fifteen global assessments from the issue survey included lists of such indicators [17,24,37–50], and these were supplemented with indicators and indices from other well-known initiatives, institutions, and databases [15,21,23,68–79]. The resulting database of over 2,000 indicators was used as a pool for representing the integrated and component issues (see S5 Dataset).

### Linking Issues to Indicators

In order to assess which issues have existing metrics that could be used to measure them, it was necessary to identify the indicators that could represent each integrated issue. Most of the indicator sources identified a particular issue that each indicator is intended to represent, and these associations between verbatim issues, integrated issues, and indicators were recorded (see S5 Dataset). Yet most indicators could be used to represent multiple integrated issues, including associations that were not noted in the original source. For instance, the Global Environmental Outlook [48] identifies”global mean temperature rise” as an indicator to measure the component issue Climate Change, which was linked to the integrated issue Air & Climate; yet this indicator could also be used to measure impacts on Ecosystem Services or vulnerability to Water Scarcity. To fully represent this multi-faceted network, these multi-issue linkages for each indicator were identified as either "related” or “fully-covering”.

#### Indicators that are related to each issue

Related indicators are defined as any indicator that can provide useful information about a given issue. Every potential link between a given indicator and the integrated and component issues was assessed by asking, “can this indicator provide useful information about this issue?”. If so, a link was made denoting that this indicator and issue are related (see S6 Dataset). All 2000+ indicators were assessed in this fashion.

#### Indicators that fully cover each issue

Fully-covering indicators are defined as any indicator that can fully represent a given issue. In other words, an indicator can be both related to an issue and fully cover an issue, but if it is not related to an issue then it cannot be fully-covering. For example, consider again “global mean temperature rise.” This indicator can provide useful information about the integrated issue Air & Climate, but it does not fully cover it, for there are many other components of Air & Climate, such as Air Pollution, for example, that cannot be measured by this one indicator. However, it does fully cover the single component issue Climate Change.

Once again, every potential link between a given indicator and the integrated and component issues was assessed by asking, “can this indicator fully cover this issue?”. If so, a link was made denoting that this indicator partially or fully covers this issue (see S7 Dataset). This process resulted in the network of issues and indicators, denoting which indicators both fully cover and are related to each integrated and component issue (see Fig. A in S1 File and S9 Dataset).

### Gap Analysis

This network of issues and indicators provided the data necessary to perform a gap analysis that identifies the issues that are the least mentioned and measured. The issues that are least mentioned by perspective were determined by calculating the percentage of the surveyed initiatives, food companies, and livelihood studies that mentioned each integrated issue. For instance, 7 of the 15 global initiatives mentioned safety (47%) compared to 9 of the 10 food companies (90%) and 3 of the 12 livelihoods studies (33%). The weighted average across all perspectives was also calculated, correcting for the difference in the number of communications surveyed from the three perspectives.

Similarly, for each communication surveyed, the percentage of issues mentioned in each capital group was calculated, along with the average for each capital group by perspective. For instance, COSA [37] mentions two-thirds of the human issues but only 45% of the social and political issues, while the average across all global initiatives surveyed is only 38% of human issues but 65% of social and political issues.

Issue measurement gaps were also assessed by identifying the integrated issues with few related indicators. First, the number of indicators related to each integrated issue was calculated. Yet having a low number of related indicators is by itself not enough to judge whether an issue has been given sufficient attention by the sustainability community. Few related indicators may simply mean the integrated issue is more narrowly defined (i.e. few component issues), and hence fewer indicators are necessary to represent it. Therefore, the number of fully-covering indicators for each component issue was also calculated. It is assumed that indicators fully covering a component issue also cover the portion of the integrated issue of which it is a component. In this way, the percentage of each integrated issue that is covered by indicators was determined as well.

### Stakeholder Validation

In order to confirm the legitimacy and usefulness of our list of integrated issues and their placement within the two conceptual frameworks, the project team convened a meeting of 20 stakeholder advisors from around the globe. Stakeholders were selected that represent divergent perspectives in the global food system, particularly those associated with our top-down public perspectives (government development agencies and policy funders), top-down private perspectives (food manufacturers, suppliers, and financial services), and bottom-up livelihood perspectives (farmers and ranchers). Other stakeholders included sustainability practitioners from environmental and social advocacy organizations commodity certifiers, and researchers. Stakeholders were also selected to represent a diverse set of regions around the world, with advisors from four continents represented overall. See Table C in S1 File for these details.

At the meeting, this advisory group was presented with the integrated issue list and asked for feedback about the scope and coverage of the issues as well as their distribution among the two frameworks and four capital groups. Note that the main goal of meeting with stakeholder advisors was to validate the integrated issue list, and hence it took place before completion of the indicator linking and the gap analysis. The results of the gap analysis from this study will be useful in further conversations with this group.

## Results

### Integrated issues by framework and organized by capital group

**Fig 1:**
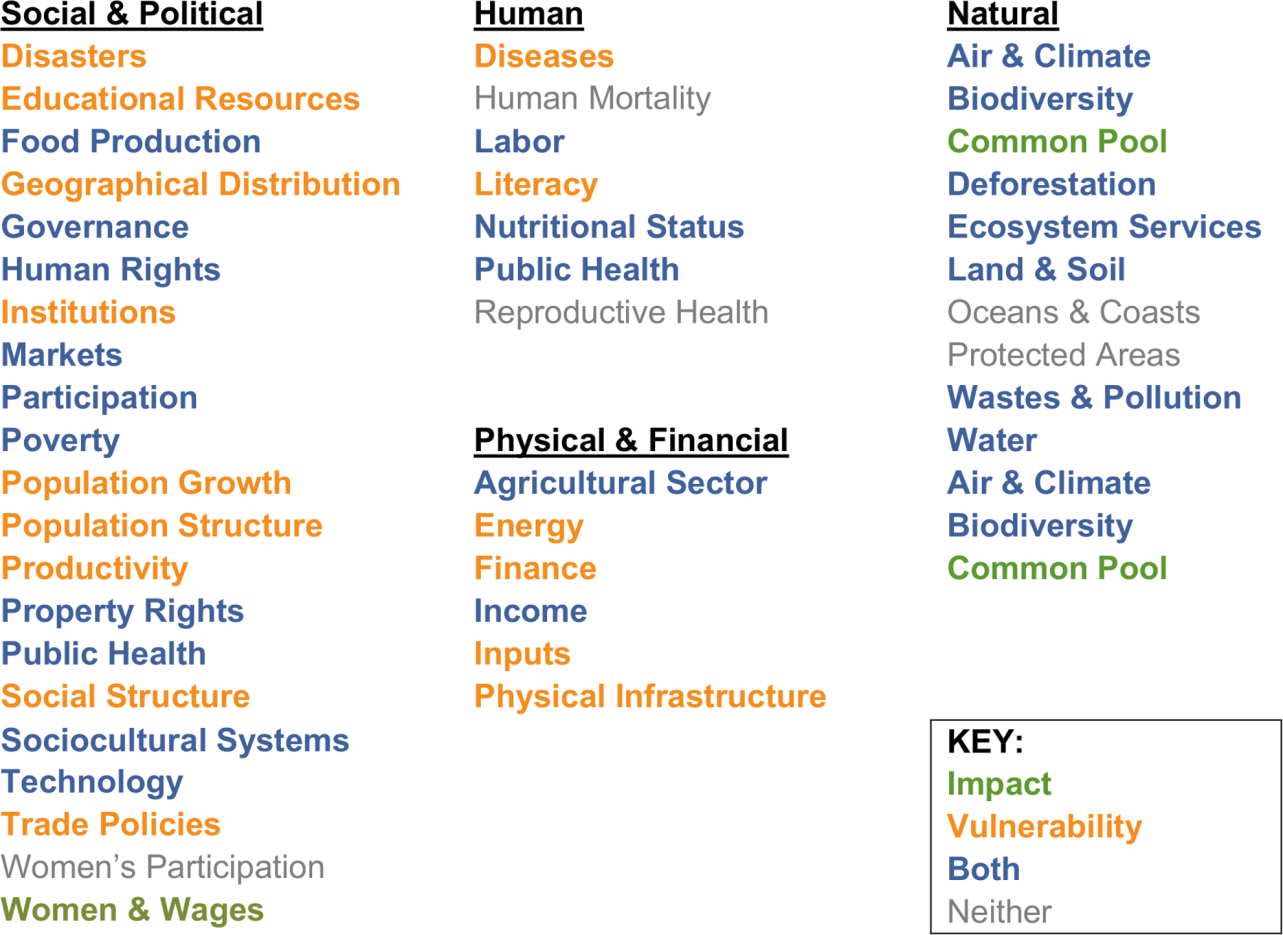
Forty-four integrated sustainability issues (24 impact and 36 vulnerability). See Information B in S1 File for detailed descriptions of each integrated issue.

Integrating the issues from different perspectives resulted in 8 human issues, 10 natural issues, 6 physical/financial issues, and 21 social/political issues for a total of 44 integrated issues (Fig 1). See Information B in S1 File for detailed definitions of each integrated issue. By framework, 24 of these issues can be impacted by sustainable sourcing decisions and 36 can make agricultural raw material sourcing more vulnerable. Fig 1 also shows that half of the issues are in both the impact and vulnerability frameworks, while five are in neither framework.

Feedback from the stakeholder engagement meeting confirmed both the usefulness of these lists of issues and frameworks in addressing sustainability and the absence of major gaps in issue coverage. In other words, the stakeholders found the list of 44 integrated issues to be comprehensive enough to serve as a defensible claim of a robust global definition of sustainability and the placement of these issues within two frameworks adequate to define the impacts and vulnerabilities of sourcing agricultural raw materials. In fact, the group recommended only one substantive change–it recommended that the integrated issue Biodiversity should be within the vulnerability framework in addition to the impact framework.

### Most and least mentioned integrated issues

**Fig 2:**
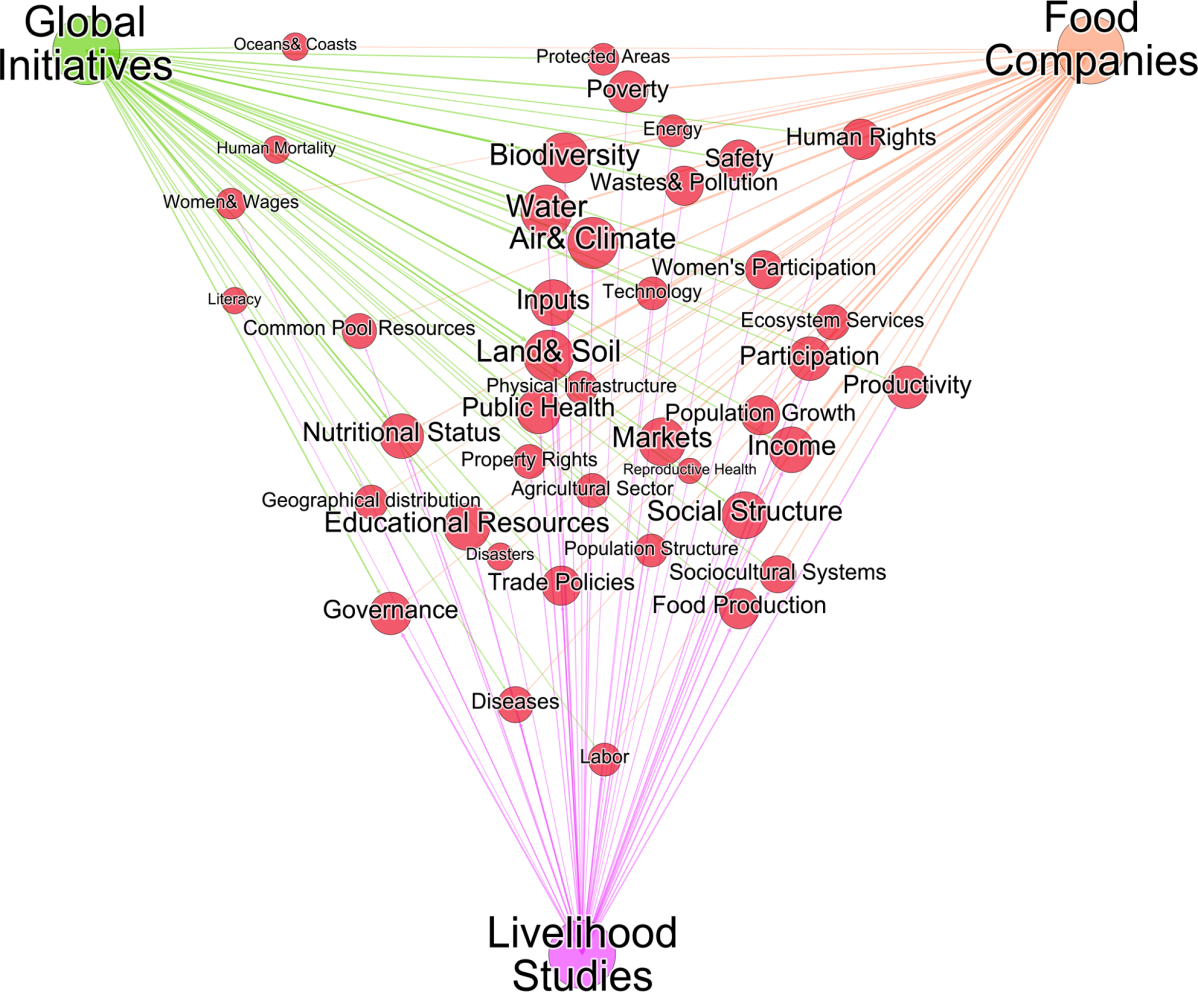
Integrated issues linked to sources by perspective. Each link represents an individual source that mentions the issue. Size of node (and text) corresponds to the number of links. Issue nodes are distributed using a force-directed algorithm (Force Atlas 2 using Gephi 0.8.2) and hence closest to perspectives with which they share the most links. See S3 Dataset.csv for data on each individual source and their issue links.

The gap analysis confirmed that prominence of particular sustainability issues varies across perspectives. Fig 2 shows the linkages between the three perspectives and the integrated issues, with each link denoting the issue is mentioned by one of the global assessments, food companies, or livelihoods frameworks. Large and central issues are often and consistently mentioned across perspectives (for instance, Land & Soil) while smaller and peripheral issues are mentioned less frequently and come primarily from individual perspectives (for instance, Labor). The relative positions of issues do not reflect the focus or priorities of individual global assessments, food companies, or livelihoods frameworks, but do indicate the issues that are of more concern to one perspective versus another.

**Fig 3:**
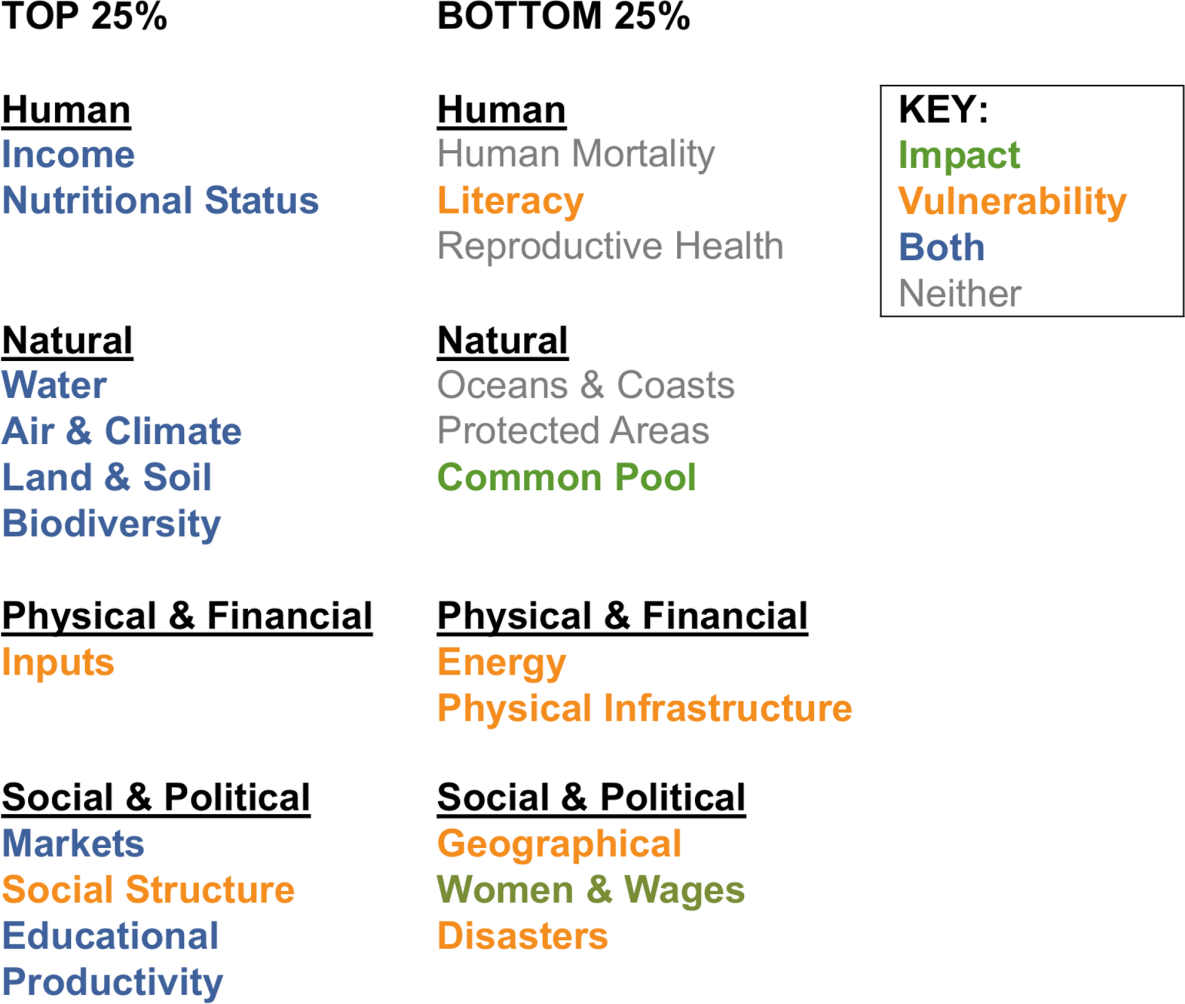
Most and least mentioned Integrated Issues across perspectives (top and bottom 25%).

The most mentioned issues across perspectives are shown in Fig 3 (those mentioned in more than 75% of all communications). The top four mentioned issues are all natural issues: Water, Air & Climate, Biodiversity, and Land & Soil. The remaining top issues from the other three groups are as follows: Markets (71%) for social/political issues, Income (66%) for human issues, and Inputs (62%) for physical/financial issues. All but three of these top issues are both impact and vulnerability issues, meaning that most of the issues addressed across perspectives are important for both frameworks.

Fig 3 also shows the least mentioned issues (those mentioned in less than 25% of all communications). These issues can be viewed as gaps that are given the least attention across perspectives. The three least mentioned issues are all human issues: Reproductive Health (8%), Literacy (9%), and Human Mortality (11%). The issues with the lowest mentions for the remaining three groups are as follows: Oceans & Coasts (15%) for natural issues, Disasters (16%) for social/political issues, and Energy (27%) for physical/financial issues. Unlike the most mentioned issues, none of the least mentioned issues are in both frameworks: half are vulnerability only, one is impact only, and the remaining are in neither framework. The least mentioned vulnerability and impact issues are Literacy and Women & Wages (22%), respectively.

**Fig 4:**
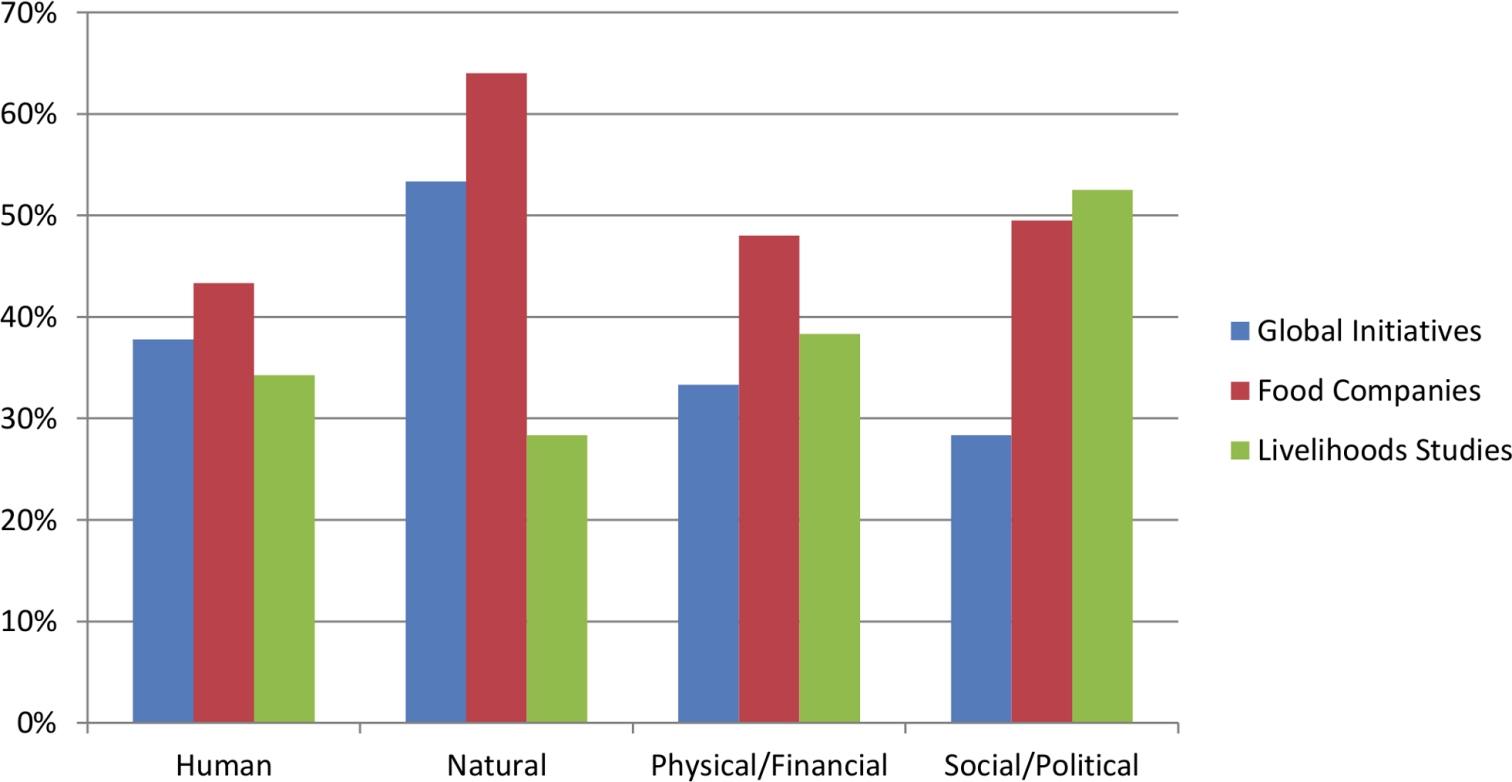
Percentage of integrated issues considered by each perspective. Organized sequentially by capital group. Percentage is an average all sampled documents and communications from all three perspectives. Note: Many livelihoods frameworks treat capital groups themselves as very broad issues, and these are not included in this figure. If counted, the breakdown of capital group mentions from the livelihoods perspective is Human (42%), Natural (83%), physical/financial (66.6%), social/political (75%), showing much higher coverage across capital groups, particularly for natural issues.

On average across perspectives, natural issues get mentioned the most (48%) and human issues the least (38%) (Fig 4). No perspective mentions more than 60% of the issues in any capital group. Both global assessments and food companies mention natural issues the most, while livelihoods frameworks mention social/political issues the most. Conversely, global assessments mention social/political issues the least, food companies mention human issues the least, and livelihoods frameworks mention natural issues the least. One stipulation: livelihoods frameworks also often mention the capital groups explicitly. If these are also counted, natural issues get mentioned the most and human the least (with all capital groups mentioned more than in either of the other two perspectives, see Fig 4 legend).

### Issues with Indicator Gaps

**Fig 5:**
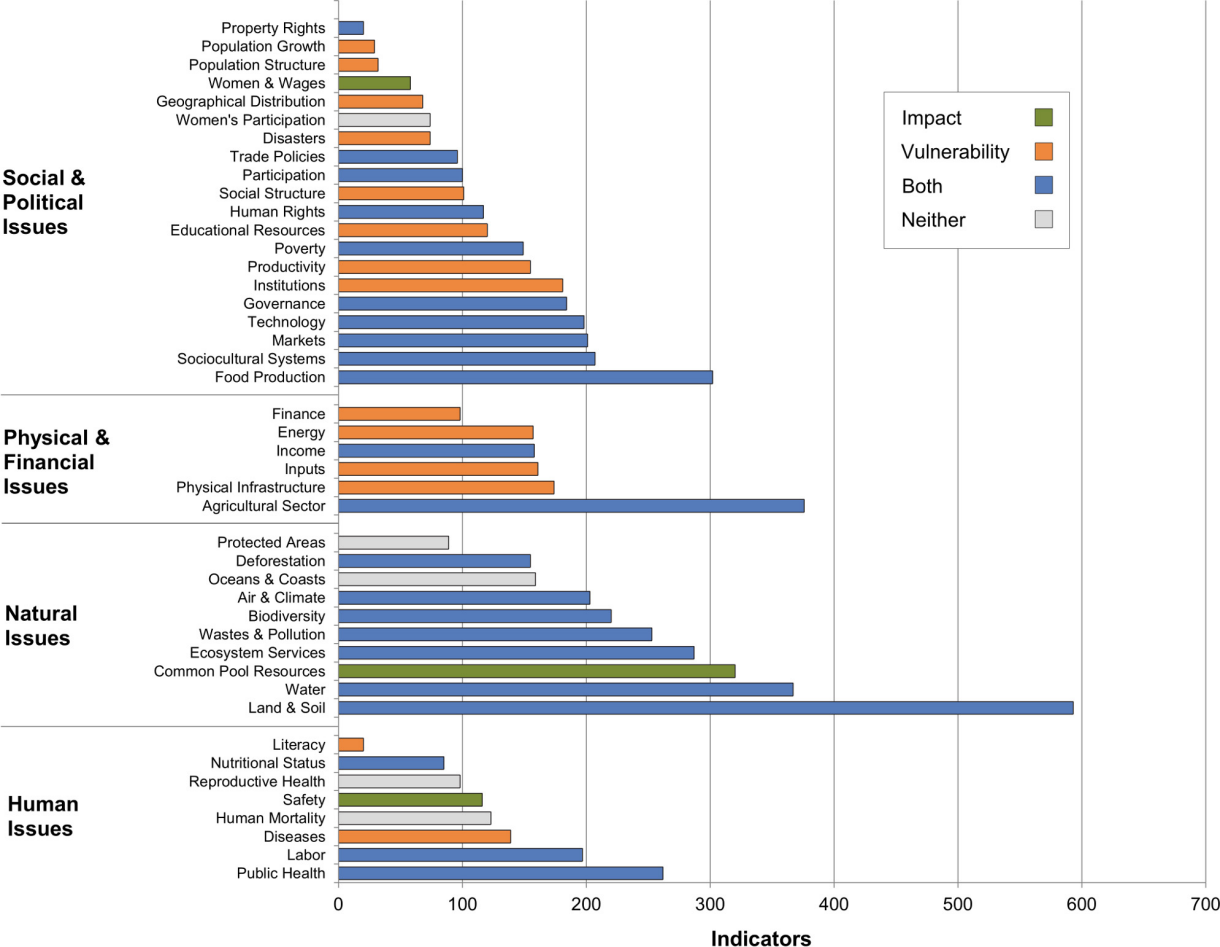
Number of related indicators per integrated issue.

All integrated issues have indicators related to them (Fig 5) and the median number of indicators related to each integrated issue is 155. Yet there is a large variance between issues: five issues (three of which are natural issues) have over 300 related indicators while thirteen issues (eight of them social/political issues) have less than 100 related indicators. Furthermore, comparing frameworks, two out of three impact issues are above the median of 165 while vulnerability issues are distributed evenly (half above, half below).

**Fig 6:**
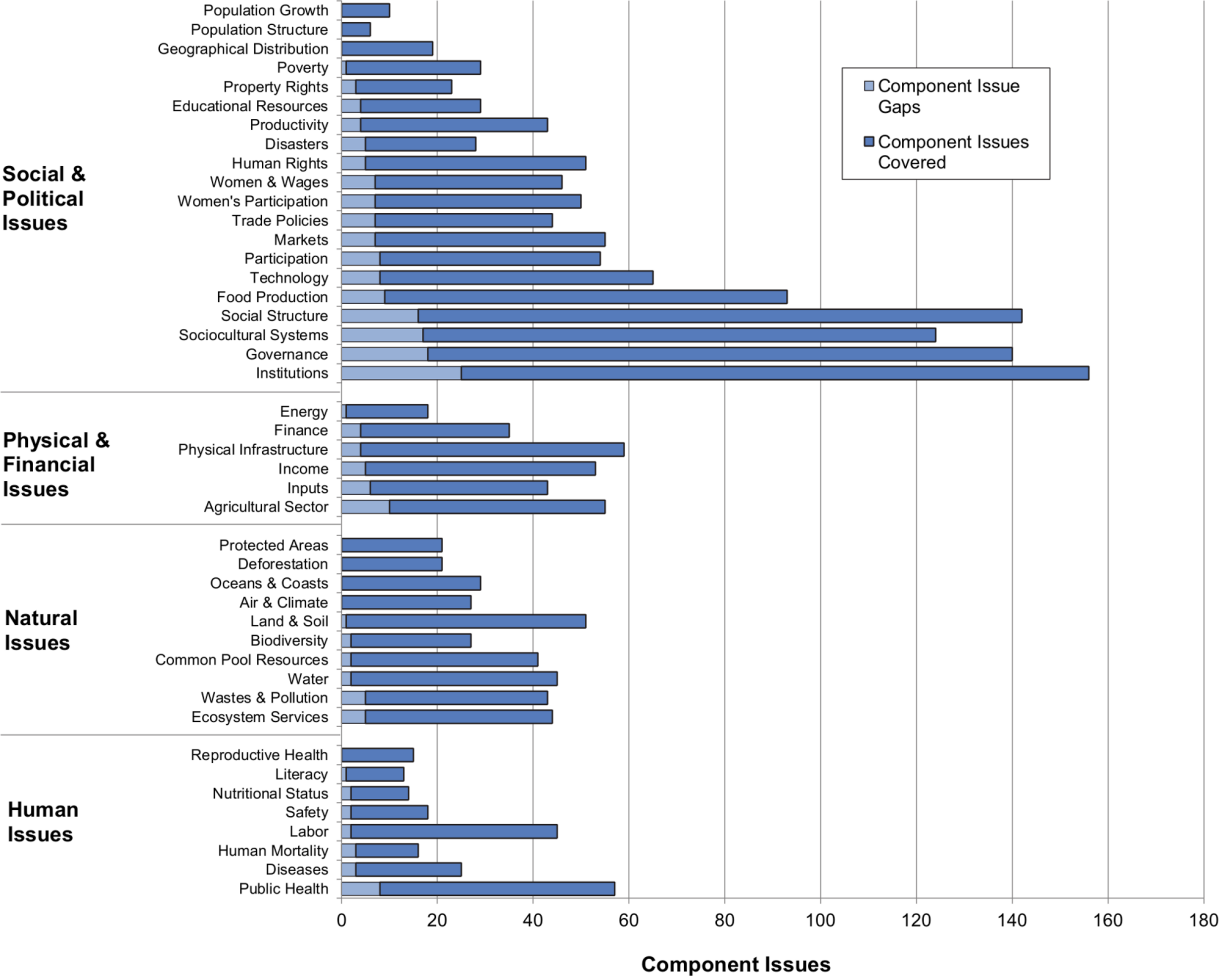
Number of fully-covered component issues for each integrated issue. See S4 Dataset.csv for a full list of component issues.

In total, 318 component issues are defined that span the 44 integrated issues (Fig 6). When the number of indicators linked to each component issue is considered, the mean number of fully-covering indicators per component issue is 3.2 indicators, and the median is 2.5 indicators. More than half of the impact component issues (59%) are above this median while vulnerability component issues are distributed about evenly (half above, half below), a similar result to the integrated issues. Of these 318 component issues, only 36 component issues (11%) do not have indicators available to fully cover them. Most of these 36 appear in the vulnerability framework (86%), while only half appear in the impact framework (53%).

These 36 “gap” issues are components of multiple integrated issues, and since it is assumed that an indicator fully covering a component issue also covers a portion of any integrated issues to which the component issue is linked, the missing indicators affect some integrated issues and capital groups more than others (Fig 6). Four of the top five affected integrated issues are social/political (Institutions, Governance, Sociocultural Systems, and Social Structure). Only eight integrated issues (18%) are unaffected by these 36 component issue gaps: four are natural issues (Air & Climate, Deforestation, Oceans & Coasts, and Protected Areas), three are social/political issues (Population Structure, Population Growth, and Geographical Distribution), and one is a human issue (Reproductive Health).

An important caveat: note in Fig 6 that the four integrated issues with the most gaps also have the most component issues that need to be covered. In fact, all integrated issues have at least 80% of their component issues covered by indicators, regardless of the number of total component issues. This suggests that the integrated issues with the most gaps may be those with relatively few indicators compared to the number of component issues they need to cover. For instance, although Social Structure has a high number of related indicators (101), it has even more component issues (120); conversely, Air & Climate–one of the eight integrated issues with fully-covered component issues–not only has twice as many related indicators as Social Structure but also five times fewer component issues.

**Fig 7:**
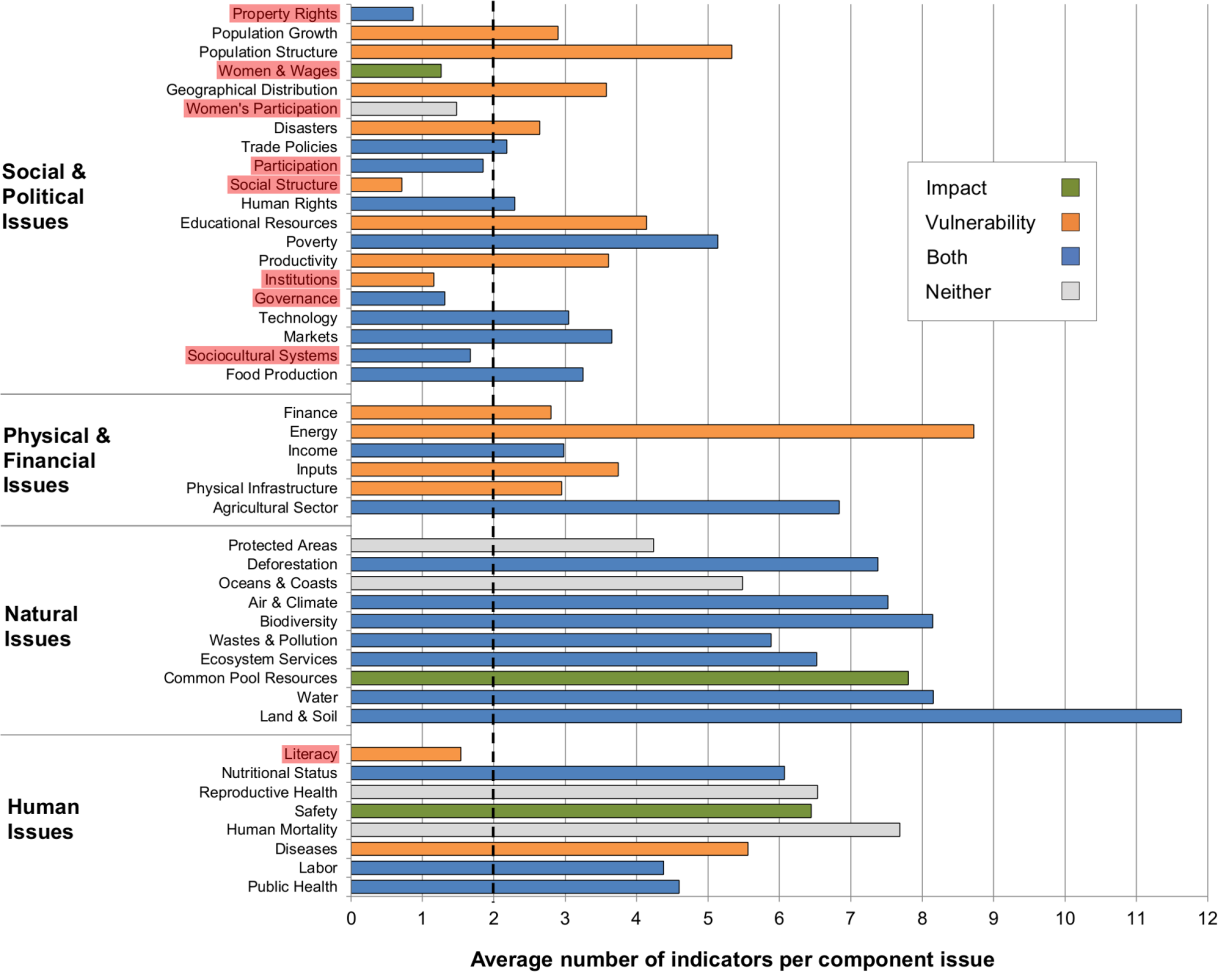
Average number of indicators per component issue (by integrated issue). A lower average suggests a lack of indicators available to fully cover a given integrated issue. Integrated issues with fewer than two indicators (on average) per component issue are highlighted in red. This threshold of two indicators (on average) per component issue is notated by the dotted line.

The average number of related indicators per component issue (Fig 7) gives a clearer picture of indicator gaps for each integrated issue. The four integrated issues with the most missing component issues in Fig 6 (including the Social Structure) also have some of the lowest indicator averages in Fig 7. Some additional integrated issues also have low indicator averages: Literacy, Participation, Property Rights, Women & Wages, and Women’s Participation all have (on average) less than two related indicators for every component issue. Only in Fig 7 do the gaps for these latter five issues become noticeable since they all have low numbers of both related indicators and component issues.

### Overlap of gaps: least mentioned and covered integrated issues

The three issues mentioned the least–Geographical Distribution, Women & Wages, and Literacy (Fig 3)–also have fewer than 100 indicators related to them (Fig 5). Furthermore, the average number of indicators per component issue for Literacy and Women and Wages are among the lowest (Fig 7). Property Rights is another issue with some of the least available indicators, few indicators per component issue, and also a relatively low number of mentions across perspectives (32%).

## Discussion

The advantage of using multiple perspectives to identify a comprehensive list of sustainability issues becomes clear through the gap analysis. As surmised, food companies tend to emphasize natural issues while livelihoods frameworks tend to emphasize social/political issues (along with very strong conceptual emphasis on capital groups as a whole, particularly natural capital, see Fig 4 legend). Global assessments do mention more issues as a whole than the other two perspectives, yet this perspective is not as balanced as hypothesized: natural issues are mentioned 15–20% more often than other capital groups. In fact, corporate and livelihoods perspectives are no less balanced that the global assessments, with at least 30% of issues mentioned for each group and a difference of no more than 25% between capital groups. This suggests that each perspective is aware of the different sustainability types and chooses issues that cut across environmental, economic, and social boundaries.

Still, although issues from all capital groups are mentioned in each case, no perspective mentions more than two-thirds of the integrated issues in each group. If all three perspectives are considered together, no issue is mentioned in more than 80% of the communications. Some of the least mentioned issues, such as Literacy and Protected Areas, are not mentioned at all by some perspectives. These gaps suggest that by considering all perspectives more issues are included than would be if each perspective were considered by itself, and as a result a more comprehensive issue list is obtained.

Without multiple perspectives, these important issues may not have been included in the master list of 44 integrated issues. Many of the oft-mentioned issues get plenty of coverage for good reason: they have been the focus of sustainability efforts for decades. For instance, natural issues such as Air and Climate, Water, and Land & Soil are synonymous with early conceptualizations of environmental sustainability as depletion of natural resources and biophysical limits to growth [80–83]. Yet the definition of sustainability is always being refined and expanded [84], and using different perspectives helps to ensure that any emerging issues are included [85]. What may seem to be an obvious and well-studied issue from one perspective may in fact be a poorly represented or measured issue from another perspective, and integrating issues helps communicate a comprehensive set of current sustainability issues across stakeholder groups that everyone should, at the very least, be aware of when defining sustainability for any particular case.

Using indicator links to find issue gaps produces similar conclusions. The existing set of available indicators has very good overall coverage across capital groups: all integrated issues have at least 20 indicators related to them, and only 36 component issues (11%) lack indicators to fully cover them. Furthermore, each integrated issue has at least 80% of its component issues fully covered by indicators. Yet as hypothesized, natural issues tend to have the most indicators while social/political issues tend to have fewer indicators (both related and fully-covering). Again, this is not particularly surprising since natural issues have a physical basis that is conducive to measurement [30,31]. And given early conceptualizations of sustainability have a strong environmental focus [9–11], social/political issues may not only be conceptually harder to measure but, until recent years, historically outside the purview of the sustainability community. As such, future public and private sustainability initiatives would do well to complement their emphasis on natural issues with robust efforts to develop and adapt metrics that assess social/political issues. For some actors, such comprehensive emphasis is already evident. For instance, the FAO Sustainability Assessment of Food and Agriculture Systems (SAFA) [50] and Unilever [7] both mention a higher percentage of social/political issues than natural issues.

This gap analysis further reveals that vulnerability issues have more gaps than impact issues. For example, Disasters, Energy, and Literacy are the least mentioned issues and each is in the vulnerability framework. Furthermore, as hypothesized, impact issues on average tend to have more indicators available to represent them, both when considering related indicators and fully-covering indicators. This suggests that additional effort should be focused upon developing indicators that can measure vulnerabilities in addition to impacts. That being said, both frameworks have issues that are rarely mentioned across perspectives: of the three issues with the largest issue *and* indicator gaps, one is an impact issue (Women & Wages), one is a vulnerability issue (Literacy), and one is both (Property Rights), so this additional effort should be targeted at specific issues as well as general categories of issues.

The suggestion that different perspectives give attention to issues outside their traditional realm of coverage does not necessarily mean that it is wrong for them to focus on particular issues or particular capital groups. In fact, it makes sense that each perspective–or even more narrowly, each particular organization or actor–would focus on a particular subset of issues that make sense from their perspective [85]. omparing issues across perspectives is a bit like comparing apples and oranges, with each perspective focused upon specific issues and ideas that are important to that particular perspective and the viewpoints and goals it represents. For instance, those actors from the livelihoods perspective may concentrate upon social/political issues more than the other two perspectives because they want to highlight critical drivers that are particularly relevant to them, such as food insecurity.

At the same time, we argue that every actor should at least be aware of all sustainability issues. Even if an issue cannot be explicitly addressed from a given perspective, general awareness of all issues is an important aspect of making truly responsible and comprehensive sustainable sourcing decisions, particularly for decisions or strategies that require stakeholder collaboration across perspectives. For instance, those actors taking the livelihoods perspective may be trying to highlight that not only are social/political issues particularly important from their perspective, but that to adequately address them they need input and collaboration from, for example, policy makers or government institutions. Such transparent communication between these two groups could be particularly valuable during a policy process to identify the key issues and strategies to achieve more sustainable food production, creating a comprehensive basis for action while also building partnerships among the actors needed to enable positive change.

The idea suggested here is that regardless of perspective, a given subset of issues should fit into and be consistent with the comprehensive list of integrated issues. Such consistency enhances the argument of a given set of actors to focus on a subset of issues, for they can say “here are the other issues that matter for sustainability in other cases, but only this subset of issues matters in our case.” And by using the same comprehensive list with the same language as other perspectives, actors can more easily communicate their chosen subset to actors from other perspectives and explain why they may not be including other issues outside the scope of their organization. Through such comparisons, actors may also find one or two issues that have been overlooked and that *do* matter for their particular case, helping refine their assessment and ensuring that important issues are not dismissed.

In fact, in conjunction of with the development of the issue and indicator network presented in this study, additional tools have been conceived that encourage and facilitate the selection of issue and indicator subsets for specific users in specific cases. At its current scope of 44 integrated issues, 318 component issues and 2000+ indicators, it remains practically difficult for stakeholders to consider, compare, and choose a reasonable set of indicators for all these issues. Corresponding research has led to the development two optimization approaches–one using the heuristic model MARXAN [86] and the other using the exact linear programming model–that can be used to help choose a small set of indicators to represent a diverse set of issues [87]. Parallel research has also produced a prototype GIS platform for viewing spatial data associated with the selected issues and indicators. We have also published a searchable database of the quotes from the public communications of food companies that were used to derive the integrated issues. This information can be sorted by issue, communication type, and industry alliance so that other actors can see which issues are actually being addressed by companies instead of simply being discussed (see Information B in S1 File). These tools are a first step toward a decision-support platform that can utilize the information in the linked network of issues and indicators.

Our team has been selective in the framing of the issues and the inclusion of indicators, yet this present network is also open to issue and indicator additions and updates as new information become available and as the science advances [36]. One way to do this is to make this information available to the global “Semantic Web”, which is a global system of semantically consistent information on the World Wide Web (Information A in S1 File). The issue and indicator network presented in this study is currently being transferred to a “wiki” platform, much like Wikipedia, but with the capability to connect with other information sources using the same controlled vocabularies such as AGROVOC. By connecting to this Semantic Web using such a wiki platform, our indicator and issue network can more easily link with other information sources while also allowing stakeholders to refine issues, update indicators, and add potential datasets among other pieces of information. This study is based upon an open-source philosophy in which transparent collaboration across perspectives is essential for addressing the gaps highlighted by this research as well as uncovering and communicating new ones, and an open and easily accessible global system of information can facilitate a convergence of sustainability definitions and measurements across stakeholder perspectives and applications.

Another next step is to consider the usefulness of each indicator given the context of its specific application (e.g. the scale of analysis, supply chain to be analyzed, availability of data, etc.). As these parameters change, new issue gaps will undoubtedly arise while others will be bridged. This current network of carefully selected issue and indicators forms a basis for an evolving, dynamic network informed by input from stakeholder partners on the relevant issues and useful, salient, and transparent indicator sets. Once linked with available datasets and modeling tools, such a network can further support informed sustainable sourcing decisions by identifying areas in sourcing programs where information is the most or least available and where the tradeoffs exist between issues that are most impacted and the greatest sources of vulnerability.

## Supporting Information

S1 File Includes a description of the organization of sustainable sourcing information as a graph database for the Semantic Web and further information regarding data availability, process documentation, and the stakeholder meeting. (DOCX)

S1 Dataset Spreadsheet presenting the verbatim issues harvested from global initiatives. (CSV)

S2 Dataset Spreadsheet presenting the verbatim corporate communications. (CSV)

S3 Dataset Spreadsheet presenting the integrated issues represented by each communication. (CSV)

S4 Dataset Spreadsheet presenting the component issues and their links with integrated issues. (CSV)

S5 Dataset Spreadsheet presenting the indicators collected for this study. (CSV)

S6 Dataset Spreadsheet presenting the indicators that are related to each issue. (CSV)

S7 Dataset csv: Spreadsheet presenting the indicators that fully-cover each issue. (CSV)

S8 Dataset Ontology file defining the relationships between issues and indicators. (OWL)

S9 Dataset Relational database containing the linked network of issues and indicators. (RDF)
