# Supplementary material for: Sustainable Sourcing of Global Agricultural Raw Materials: Assessing Gaps in Key Impact and Vulnerability Issues and Indicators
Source: PLoS One. 2015 Jun 11;10(6):e0128752. doi: 10.1371/journal.pone.0128752 (PMC4465747; doi:10.1371/journal.pone.0128752)
Supplement: S1 File — (DOCX) [file pone.0128752.s001.docx]

**S1 File**

**Sustainable sourcing of global agricultural raw materials: assessing gaps in key impact and vulnerability issues and indicators**

Authors: Nathaniel P. Springer^1^*, Kelly Garbach^1^, Kathleen Guillozet^1^, Van R. Haden^1^, Prashant Hedao^2^, Allan D. Hollander^2^, Patrick R. Huber^2^, Christina Ingersoll^1^, Megan Langner^1^, Genevieve Lipari^1^, Yaser Mohammadi^1^, Ruthie Musker^1^, Marina Piatto^1^, Courtney Riggle^1^, Melissa Schweisguth^1^, Emily Sin^2^, Sara Snider^1^, Nataša Vidic^1^, Aubrey White^1^, Sonja Brodt^1^, James F. Quinn^2^, Thomas P. Tomich^1^

**1** Agricultural Sustainability Institute, University of California Davis, Davis, California, United States of America **2** Information Center for the Environment, University of California Davis, Davis, California, United States of America

**Information A. Description of sustainable sourcing information organized as a graph database for the Semantic Web**

The network of issues and indicators created for this study has been organized in a graph, or a network composed of nodes and edges. The issues and indicators are defined as nodes while the linkages between them are the edges. An example of indicator-linkage-issue (or node-edge-node) is “Global Mean Temperature Rise can provide useful information about Air & Climate” while an example of a perspective-linkage-issue is “Unilever mentions Air & Climate.” Once multiple linkages are made, the resulting graph can be visualized as a network diagram. Fig. 2 in the main paper is an example of a network diagram between perspectives, mentions, and issues (with the individual documents from each perspective aggregated).

The issue-indicator network diagram is shown in Fig. A. In the case of these relationships, each node-edge-node is also a subject-predicate-object: the indicator is the subject, the relationship is the predicate, and the issue is the object. Such graph relationships can be stored in a database format known as a “triplestore”, in which the subjects, predicates, and objects are represented in three separate columns in a table. Once encoded in triplestores, one can search and query the linked data using a language called SPARQL [1], visualize the data in networks with software such as Gruff [2], and ultimately link the data with other organizations that have also stored their information in triplestores.

This latter point is essential and known as the Semantic Web. Declaring our vocabularies using Semantic Web mechanisms is what ultimately allows our team to link our issue-indicator network with the global community. The language we have used to define our integrated and component issues comes primarily from controlled vocabularies that were created by the FAO [3] and Library of Congress [4] to make consistent subjects and object terms. By using these terms, our information is accessible to the broader linked open data community [5]. And by adding our own terms to supplement current vocabularies, we contribute the expansion of sustainable agriculture and sourcing linked data.

Using these semantic technologies, our research team intends to enable a dynamic network of sustainable sourcing information for agricultural raw materials.

**Information B. Additional description of data availability, process documentation, and stakeholder meeting**

The data collected, organized, and created for this study are made available as organized datasets to maintain transparency, assure reproducibility of our methods, and provide data that others can use for future work. The description of this data is organized below to correspond with its presentation in the methods section of the main manuscript. The datasets themselves can be downloaded as separate SI dataset files (see below).

1. *Verbatim Issues*
   1. Global Initiatives Data

The fifteen initiatives analyzed, along with the respective reports from which the verbatim issues were harvested, are presented in Table A. The list of verbatim issues collected from these reports are available in S1 Dataset.csv.

- 1. Corporate Communications Data

The 524 corporate communications are organized in S2 Dataset.csv. Each entry contains the link to the URL that was surveyed along with a timestamp and examples from the verbatim text. Note that Kraft Foods split into two companies in 2012 after this study began and is hence represented by both Kraft Foods Group and Mondelez International. This information, along with communications from five additional global food companies, can also be perused at <http://asi.ice.ucdavis.edu/sustsource/corpcomms>.

- 1. Livelihoods Frameworks Data

Table B lists the 12 studies and frameworks that were surveyed for this perspective. Each figure and the respective verbatim issues can be found on the page number listed in the table.

1. *Integrated Issues*

The links between each integrated issue and the verbatim issues contained in the communications from the three perspectives – 15 global assessments, 10 food companies, and 12 livelihoods frameworks – are available in S3 Dataset.csv. This information is the basis Figs. 2 – 4 in the main text. Detailed descriptions of the 44 Integrated Issues available online at <http://asi.ucdavis.edu/research/ss/files/Integrated%20Sustainability%20Issue%20Descriptions%2020131003.pdf>

1. *Component Issues*

A full list of component issues along with the URI if taken from either AGROVOC (<http://aims.fao.org/aos/agrovoc/c_>) or the Library of Congress (http://id.loc.gov/authorities/subjects/) is available in S4 Dataset.csv. This dataset also describes the links to each integrated issue (with each “1” describing a link and each “0” describing no link).

1. *Indicators*

Each indicator identified and used in this study can be found in S5 Dataset.csv, including its source and location within the original document or dataset.

1. *Graph Database of Issues and Indicators*

The complete network of issues and indicators constructed to perform the gap analysis is available as semantically-enabled datasets as S8 Dataset.owl and S9 Dataset.rdf. Key pieces of this dataset that were used for the gap analysis are also provided as CSV files, including the links between each integrated issue and component issue (S4 Dataset.csv) and the links between issues and indicators, both related (Dataset S6.csv) and fully-covering (S7 Dataset.csv). Note that each link is represented with a “1” and no link is represented with a “0” (and in the case of S7 Dataset.csv, partial coverage can be represented by any number in between). The respective capital group and frameworks for each integrated issue can also be found in S6 Dataset.csv. These datasets are the basis for Figs. 5 – 7 in the main text and can be visualized in Fig. A. The complete network in S9 Dataset.rdf can also be explored through a semantic wiki available at <http://asi.ice.ucdavis.edu/sustsource/wiki/>.

1. *Process Documentation*

Our research team has compiled an extensive process documentation that provides additional detail and background of the methods described in the main manuscript. The full document is available at <http://asi.ucdavis.edu/research/ss/files/Sustainable_Sourcing_Process_Documentation_2011_0107.pdf>.

1. *Stakeholder Advisors*

A list of our stakeholder advisors and their role within the food system is presented in Table C. A detailed report of the December 2012 stakeholder meeting is also available online at <http://asi.ucdavis.edu/research/ss/stakeholder-meeting-december-2012>.

**Fig. A. Network diagram of the issue-indicator graph database.** Integrated issues (red) are linked to their respective component issues (blue). Indicators (green) are linked to both integrated and component issues if they provide any useful information about that issue. The size of indicator circles is proportional to the number of issues to which it links.

**
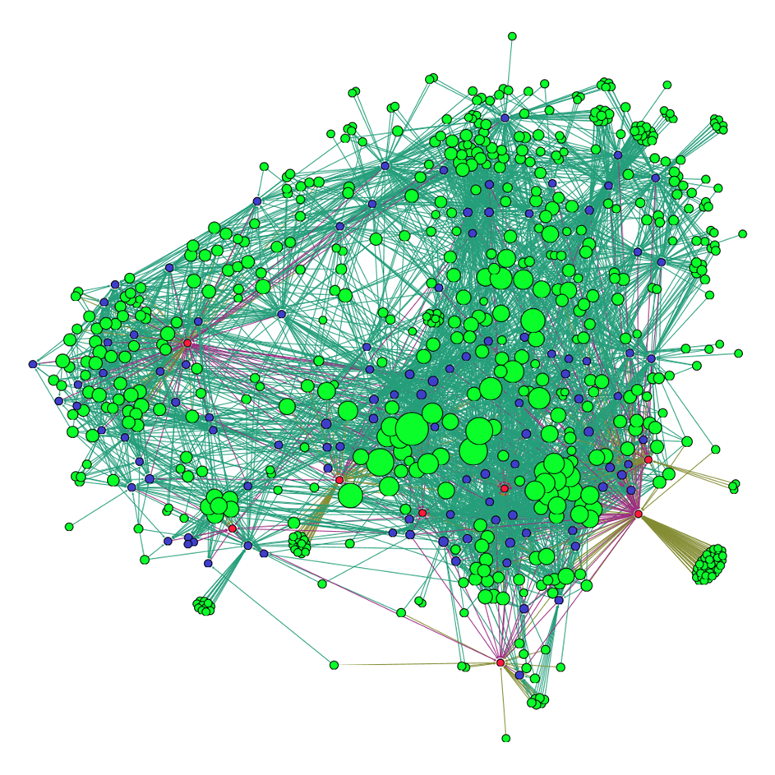
**

**Table A. Global initiatives analyzed to harvest sustainability issues**

|  | **Global Initiative [Source]** | **Organizations** |
| --- | --- | --- |
| 1 | A Conceptual Framework for Progressing Towards Sustainability in the Agriculture and Food Sector [6] | Food and Agriculture Organization and the International Social and Environmental Accreditation and Labelling Alliance (FAO-ISEAL) |
| 2 | Committee on Sustainability Assessment [7] | Committee on Sustainable Assessment (COSA) |
| 3 | Convention on Biological Diversity [8] | NASA, United Nations Environmental Programme (UNEP) |
| 4 | Global Environmental Outlook: Integrated Environmental Assessment [9] | United Nations Environmental Programme (UNEP) |
| 5 | Global International Waters Assessment [10] | United Nations Environmental Programme (UNEP) |
| 6 | Global Reporting Initiative [11] | Global Reporting Initiative (GRI) |
| 7 | Indicators of Sustainable Development [12] | United Nations Commission on Sustainable Development |
| 8 | International Assessment of Agricultural Knowledge, Science and Technology for Development [13] | World Bank, Food and Agriculture Organization, Others |
| 9 | Land Degradation Assessment in Drylands [14,15] | Food and Agriculture Organization (FAO) |
| 10 | Millennium Development Goals [16] | United Nations |
| 11 | Millennium Ecosystem Assessment [17] | Millennium Ecosystem Assessment |
| 12 | Putting the New Vision for Agriculture into Action [18] | World Economic Forum (WEF) |
| 13 | State of Sustainability Initiatives [19] | Sustainable Commodity Initiative |
| 14 | Sustainability Assessment of Food and Agriculture (SAFA) [20] | Food and Agriculture Organization (FAO) |
| 15 | World Water Development Report [21] | United Nations Educational, Scientific, and Cultural Organization (UNESCO) |

**Table B. Livelihoods frameworks analyzed to harvest sustainability issues**

|  | **Authors [Source]** | **Pg. #** | **Title (Figure)** |
| --- | --- | --- | --- |
| 1 | Duhaime and Godmaire [22] | 93 | Food System (Figure 1) |
| 2 | Ecker and Breisinger [23] | 5 | Conceptual framework of the food security system (Figure 2.1) |
| 3 | Ericksen [24] | 6 | Food systems and their drivers (Figure 1) |
| 4 | Jonsson [25] | 11 | Causes of Malnutrition (Figure 1) |
| 5 | Moser and Norton [26] | 18 | Human Rights and Sustainable Livelihoods (Table 1) |
| 6 | Pender et al. [27] | 32 | Factors Affecting Income Strategies, Land Management, and Their Implications (Figure 2.1) |
| 7 | Riely et al. [28] | 13 | Food Security Conceptual Framework (Diagram 2) |
| 8 | Scoones [29] | 4 | Sustainable Rural Livelihoods (Figure 1) |
| 9 | Sobal et al. [30] | 857 | Food and Nutrition System (Figure 3) |
| 10 | Soussan et al. [31] | 8 | Livelihoods Model (Figure 1) |
| 11 | Stamoulis and Zezza [32] | 8 | Food and Nutrition Security (Figure 1) |
| 12 | Yu et al. [33] | 59 | Food Systems and Their Interaction with Global Change (Figure 1) |

**Table C. Stakeholder representatives and their role within the food system.** All participants were partners in preparing and convening a meeting in December 2012 to discuss the coverage and usefulness of the 44 integrated issues and how they fit into the two frameworks.

|  | **Organization** | **Food System Role** |
| --- | --- | --- |
| 1 | Maplecroft | Research & Assessment |
| 2 | Global Environmental Facility | Policy Funding Agency |
| 3 | Royal Government of Bhutan | Government Development Agency |
| 4 | Rabobank International | Financal Services |
| 5 | Harvard University | Research & Assessment |
| 6 | Mars Incorporated | Food Manufacturer |
| 7 | Oxfam America | Environmental & Social Advocate |
| 8 | Solutions for the Land | Farmer/Rancher |
| 9 | UC Davis | Research & Assessment |
| 10 | Bunge | Food Commodity Supplier |
| 11 | Colorado State University | Research & Assessment |
| 12 | Ladder Livestock | Farmer/Rancher |
| 13 | Greenpeace International | Environmental & Social Advocate |
| 14 | World Resources Institute | Environmental & Social Advocate |
| 15 | Sugarcane Growers Assn. of Capivari | Farmer/Rancher |
| 16 | Mars Incorporated | Food Manufacturer |
| 17 | IMAFLORA | Certifier |
| 18 | IFPRI | Research & Assessment |
| 19 | Mars Incorporated | Food Manufacturer |
| 20 | Campbell Soup Company | Food Manufacturer |

**References**

1. W3C SPARQL Working Group. SPARQL 1.1 overview [Internet]. 21 Mar 2013 [cited 13 Jan 2014]. Available: http://www.w3.org/TR/sparql11-overview/

2. Aasman J, Cheetham K. Rdf browser for data discovery and visual query building. In: Handschuh S, Aroyo L, Thai V, editors. Proceedings of the Workshop on Visual Interfaces to the Social and Semantic Web. Palo Alto, CA: CEUR Workshop Proceedings; 2011. pp. 1 – 4. Available: http://ceur-ws.org/Vol-694/paper9.pdf

3. Caracciolo C, Morshed A, Stellato A, Johannsen G, Jaques Y, Keizer J. Thesaurus maintenance, alignment and publication as linked data: the agroovoc use case. In: García-Barriocanal E, Cebeci Z, Okur MC, Öztürk A, editors. Metadata and Semantic Research: Communications in Computer and Information Science. Springer Berlin Heidelberg; 2011. pp. 489–499. Available: http://link.springer.com/chapter/10.1007/978-3-642-24731-6_48

4. Library of Congress. Library of congress subject headings - LC linked data service (LIbrary of Congress) [Internet]. 2013 [cited 13 Jan 2014]. Available: http://id.loc.gov/authorities/subjects.html

5. Bizer C, Heath T, Berners-Lee T. Linked data - the story so far. International Journal on Semantic Web and Information Systems. 2009;5: 1–22. doi:10.4018/jswis.2009081901

6. Guttenstein E, Scialabba NE, Loh J, Courville S. A conceptual framework for progressing towards sustainability in the agriculture and food sector [Internet]. FAO-ISEAL Alliance; 2010 pp. 1–24. Available: http://www.fao.org/docrep/012/al322e/al322e00.pdf

7. COSA. Basic indicators for farm level [Internet]. Committee on Sustainability Assessment (COSA); 2012. Report No.: v.2.1. Available: http://thecosa.org/wp-content/uploads/2013/09/Basic-Indicators-v3-4.pdf

8. Strand H, Hoft R, Strittholt J, Miles L, Horning N, Fosnight E, et al. Sourcebook on remote sensing and biodiversity indicators [Internet]. Montreal: Convention on Biological Diversity; 2007. Available: http://cce.nasa.gov/pdfs/cbd-ts-32_sourcebook.pdf

9. Van Woerden J, Wieler C, Gutierrez E, Grosshans R, Abdelrehim A, Rajbhandari PCL. Training module 4: monitoring, data, and indicators [Internet]. UNEP; 2008. Report No.: 4. Available: http://www.unep.org/ieacp/_res/site/File/iea-training-manual/module-4.pdf

10. UNEP. Challenges to international waters - regional assessments in a global perspective [Internet]. Nairobi, Kenya: United Nations Environmental Programme (UNEP); 2006 p. 120. Available: http://www.unep.org/dewa/giwa/publications/finalreport/giwa_final_report.pdf

11. GRI. GRI G3 and G3.1 Update – Comparison Sheet [Internet]. Global Reporting Initiative; 2011. Available: https://www.globalreporting.org/resourcelibrary/G3.1-Comparison-Sheet.pdf

12. United Nations Department of Economic and Social Affairs. Indicators of sustainable development: guidelines and methodologies [Internet]. UN; 2001. Report No.: 2nd Edition. Available: http://sustainabledevelopment.un.org/content/documents/indisd-mg2001.pdf

13. International Assessment of Agricultural Knowledge, Science and Technology for Development. Agriculture at a crossroads: executive summary of the synthesis report [Internet]. Washington, D.C.: Island Press; 2009. Available: http://www.unep.org/dewa/assessments/ecosystems/iaastd/tabid/105853/default.aspx

14. Bunning S, McDonagh J, Rioux J. LADA Manual for local level assessment of land degradation and sustainable land management [Internet]. Rome: FAO; 2011. Report No.: Part 1: Planning and methodological approach, analysis and reporting. Available: http://www.fao.org/fileadmin/templates/nr/kagera/Documents/LADA_manuals/MANUAL1_final_draft.pdf

15. Bunning S, McDonagh J, Rioux J. LADA Manual for local level assessment of land degradation and sustainable land management [Internet]. Rome: FAO; 2011. Report No.: Part 2: Field methodology and tools. Available: http://www.fao.org/fileadmin/templates/nr/kagera/Documents/LADA_manuals/MANUAL2_final_draft.pdf

16. UN. Official list of MDG indicators [Internet]. 15 Jan 2008 [cited 13 Jan 2014]. Available: http://mdgs.un.org/unsd/mdg/Resources/Attach/Indicators/OfficialList2008.pdf

17. Scholes R, Biggs R, Palm C, Duraiappah A. Assessing state and trends in ecosystem services and human well-being. In: Ash N, Blanco H, Brown C, Garcia K, Henrichs T, Lucas N, et al., editors. Ecosystems and human well-being: a manual for assessment practitioners. Washington; Covelo; London: Island Press; 2010. pp. 115 – 150.

18. World Economic Forum. Putting the new vision for agriculture into action: transformation is happening [Internet]. Geneva, Switzerland: World Economic Forum; 2012. Available: http://www3.weforum.org/docs/WEF_FB_NewVisionAgriculture_HappeningTransformation_Report_2012.pdf

19. State of Sustainability Initiatives. State of sustainability preliminary list of indicators [Internet]. Sustainable Commodity Initiative; 2009. Available: http://sustainablecommodities.org/files/SSI%20Indicator%20List.pdf

20. FAO. Sustainability Assessment of Food and Agriculture Systems (SAFA) guidelines [Internet]. Rome: FAO Natural Resources Management and Environment Department; 2012. Available: http://www.fao.org/fileadmin/templates/nr/sustainability_pathways/docs/SAFA_Guidelines_12_June_2012_final_v2.pdf

21. World Water Assessment Programme. The united nations world water development report 4: managing water under uncertainty and risk [Internet]. Paris: UNESCO; 2012. Available: http://unesdoc.unesco.org/images/0021/002156/215644e.pdf

22. Duhaime G, Godmaire A. The conditions of sustainable food security: an integrated conceptual framework. Pimatziwin: A Journal of Aboriginal and Indigenous Community Health. 2002;1: 88–127.

23. Ecker O, Breisinger C. The food security system: a new conceptual framework [Internet]. IFRPI Development Strategy and Governance Division; 2012 Mar. Report No.: 01166. Available: http://www.ifpri.org/sites/default/files/publications/ifpridp01166.pdf

24. Ericksen PJ. Conceptualizing food systems for global environmental change research. Global Environmental Change. 2008;18: 234–245. doi:10.1016/j.gloenvcha.2007.09.002

25. Jonsson U. Nutrition and the united nations convention on the rights of the child [Internet]. Florence, Italy: UNICEF; 1993. Report No.: 5. Available: http://www.unicef-irc.org/publications/pdf/crs5.pdf

26. Moser C, Norton A, Conway T, Ferguson C, Vizard P. To claim our rights: livelihood security, human rights and sustainable development [Internet]. London: Overseas Development Institute (ODI); 2001. Available: http://www.odi.org.uk/resources/docs/1816.pdf

27. Pender J, Place F, Ehui S, editors. Strategies for sustainable land management in the east african highlands [Internet]. Washington, DC: International Food Policy Research Institute; 2006. Available: http://www.ifpri.org/sites/default/files/pubs/pubs/books/oc53/oc53toc.pdf

28. Riely F, Mock N, Cogill B, Bailey L, Kenefick E. Food security indicators and framework for use in the monitoring and evaluation of food aid programs [Internet]. Arlington, VA: USAID; 1999. Available: http://pdf.usaid.gov/pdf_docs/PNACG170.pdf

29. Scoones I. Sustainable rural livelihoods: a framework for analysis [Internet]. Brighton, UK: Institute of Development Studies; 1998. Report No.: 72. Available: http://www.ids.ac.uk/files/dmfile/Wp72.pdf

30. Sobal J, Khan LK, Bisogni C. A conceptual model of the food and nutrition system. Soc Sci Med. 1998;47: 853–863. doi:10.1016/S0277-9536(98)00104-X

31. Soussan J, Blaikie P, Springate-Baginski O, Chadwick M. Understanding livelihood processes and dynamics [Internet]. DFID; 2000. Report No.: 1. Available: http://www.cesca-world.org/downloads/Understanding%20Livelihood%20Processes.pdf

32. Stamoulis K, Zezza A. A conceptual framework for national agricultural, rural development, and food security strategies [Internet]. FAO; 2003. Available: ftp://ftp.fao.org/docrep/fao/007/ae050e/ae050e00.pdf

33. Yu Q, Wu W, Yang P, Li Z, Xiong W, Tang H. Proposing an interdisciplinary and cross-scale framework for global change and food security researches. Agriculture, Ecosystems & Environment. 2012;156: 57–71. doi:10.1016/j.agee.2012.04.026
